# Eyes Are Windows to the Chinese Soul: Evidence from the Detection of Real and Fake Smiles

**DOI:** 10.1371/journal.pone.0019903

**Published:** 2011-05-25

**Authors:** Xiaoqin Mai, Yue Ge, Lin Tao, Honghong Tang, Chao Liu, Yue-Jia Luo

**Affiliations:** 1 Center for Human Growth and Development, University of Michigan, Ann Arbor, Michigan, United States of America; 2 State Key Laboratory of Cognitive Neuroscience and Learning, Beijing Normal University, Beijing, China; 3 Department of Sociology, the Chinese University of Hong Kong, Hong Kong, China; University of Minnesota, United States of America

## Abstract

How do people interpret the meaning of a smile? Previous studies with Westerners have found that both the eyes and the mouth are crucial in identifying and interpreting smiles, yet less is known about Easterners. Here we reported that when asking the Chinese to judge the Duchenne and non-Duchenne smiles as either real or fake, their accuracy and sensitivity were negatively correlated with their individualism scores but positively correlated with their collectivism scores. However, such correlations were found only for participants who stated the eyes to be the most useful references, but not for those who favored the mouth. Moreover, participants who favored the eyes were more accurate and sensitive than those who favored the mouth. Our results thus indicate that Chinese who follow the typical Eastern decoding process of using the eyes as diagnostic cues to identify and interpret others' facial expressions and social intentions, are particularly accurate and sensitive, the more they self-report greater collectivistic and lower individualistic values.

## Introduction

### Smiles, real and fake smiles

The smile is the most common and consistent human facial expression. By definition, a smile is characterized by the upward turn of the corners of the lips, as being produced by the contraction of the zygomaticus major muscle [1]. Recently, one specific division between different types of smiles has received more and more attention, that is, whether a smile is real or fake [2], [3]. A real smile is the normative or default type, which means that the smile conveys positive feelings (e.g., pleasure or joy) or positive intentions (e.g., appreciation or encouragement). In contrast, a fake smile means that the smile is prompted by a desire to hide, moderate, or justify something negative (e.g., lie or criticism) [4]. Duchenne [5] and more recently Ekman and his colleagues [6] have reported morphological indicators for the real smiles. In addition to the action of zygomaticus major muscle that pulls the lip corners up, real smiles also involve recruitment of the muscles around the eyes, the orbicularis oculi, specifically the pars lateralis part (the Duchenne marker). When contracted, this muscle causes wrinkles at the outer corners of the eyes, a lifting of the cheeks, bagging or bulging of skin below the eye, lowering of the eyebrows and a narrowing of the eye opening, resulting in a real smile (Duchenne smile). In contrast, most of these features will not be presented during a fake smile [4], [7], [8].

### The role of the eyes and mouth in smiles: Western cultures

In Western cultures, the mouth is crucial in identifying and interpreting facial expressions, especially with regard to happiness and smiles. A rich body of literature supports this idea. The first piece of evidence comes from the study of patient SM, who has severely impaired identification of anger and fear after damaging the bilateral amygdala but still preserves an intact detection of happiness [9], [10]. The reason for this dissociation is because of patient SM's inability to make normal use of information from the eyes but an undamaged ability to use information from the mouth [11]. It therefore provides direct evidence that the mouth alone, through behavioral adaptation and neural plasticity, could supply sufficient information for the recognition of happiness and smiles, which has also been confirmed by the computational analysis of FACS-coded faces [12]. In addition, the mouth is also a common operation object in smile studies, e.g., in the study from Soussignan [13], smiling or not are simulated by holding a pencil in the teeth or lips, respectively.

However, interpreting the meaning of a smile also depends on the information conveyed by the eyes, especially for distinguishing between real and fake smiles. Ekman [14] noted that expressions lacking the expressive cues in the eyes are perceived as poor examples of positive emotional expressions. Frank and his colleagues [7] further showed that people can discriminate between enjoyment (or real) and nonenjoyment (fake) smiles at above chance level only when the observers specifically monitored the eye region. Furthermore, individuals showing enjoyment smiles were evaluated as more positive (e.g., expressive, sociable, relaxed) than individuals expressing nonenjoyment smiles. Williams, Senior, Loughland, and Gordon [15] further proposed that the distinction between real and fake smiles is so important that, when a smiling face is detected, attention is automatically directed towards eyes and eye corners to evaluate the authenticity of the smile. In support of this hypothesis, they demonstrated that observers make more fixations to eye corners (crow's-feet wrinkles) for smiling compared to neutral or sad faces.

### The role of the mouth and eyes in smiles: Eastern cultures

Nonetheless, most studies on smiles have been conducted in Western cultures. What about Eastern cultures? In fact, an amount of observational and experimental evidence suggests that people in Eastern cultures may evaluate the role of the mouth and eyes in smiles differently than people in Western cultures.

In Eastern cultures, especially China, “one must NOT show ones' teeth when smiling” is a strict rule of discipline for women that has lasted thousands of years, ever since the Tang Dynasty (so the Mona Lisa's smile could also have been appreciated by ancient Chinese). Ancient Chinese women even used adornments around the mouth (e.g., fake dimples) to compensate for the lack of emotional information conveyed by the mouth during their closed-mouth smiles. A good example of such an historic and prevalent influence of cultural value on the role of the mouth in smiles can be illustrated by contrasting the smile emoticons used on the Internet by Easterners and Westerners. In common Western smile emoticons such as :-) or :), the mouth is exaggerated with a crimped line whereas the eyes are simplified as two dots. As a contrast, Japanese use smile emoticons with a simplified mouth but crimped eyes, e.g., (^ʌ^.^ʌ^) or (^ʌ^_^ʌ^) [16], [17], [18]. Chinese, especially females, go even further by not only adopting simplified mouth and crimped eyes, but also inusing them with the ancient tradition of attaching fake dimples, e.g., (*^ʌ^_^ʌ^*), ( = ^ʌ^_^ʌ^ = ), or (@^ʌ^_^ʌ^@) [19]. Apparently, compared with Westerners, Japanese and Chinese people value the eyes much more highly than the mouth when they try to express their happiness in an abstract way.

Will such a cross-cultural difference between the role of the mouth and eyes in smiles found in daily life be replicated in experimental studies? The answer is yes. In two studies using either emotional expressions in emoticons or computer-edited photographs of real faces, Yuki et al [18] compared the difference between Japanese and Americans in weighing facial cues when interpreting emotional expressions. Results showed that compared to Japanese, Americans weighed expression cues displayed in the mouth more when judging emotions, whereas Japanese tended to weigh expression cues in the eyes more than Americans. More recently, in an eye-tracking study investigating the decoding of facial expression signals in a facial expression categorization task with real face pictures, Jack et al [16] found that Easterners (12 Chinese and 1 Japanese) and Westerners (13 European) adopt different decoding strategies when reading others' facial expressions. Westerners distributed their fixations evenly across the face, whereas Easterners systematically biased their fixations toward the eye region and ignored the mouth region. Moreover, Easterners constantly neglect critical aspects of FACS-coded faces defined by Westerners [12].

One interpretation for the previous cross-cultural difference on the role of the mouth is that Easterners are good at regulating facial expressions because of the restriction of expressing individual emotion in public in Eastern cultures, which is especially true for those expressions that come from the mouth [18]. This is because muscles around the mouth are much easier to operate than those around the eyes, which in general are not under voluntary control [14]. As a result, Easterners usually weigh information they read from others' mouths much less than those from others' eyes [18], [20]. In addition, studies have found that individuals from Eastern cultural contexts (Japanese) understand emotions as arising in the relations between people (collectivistic), whereas those from Western cultural contexts (American) understand emotions as arising primarily within people (individualistic) [21]. These explanations and results together have an important implication, that is, individuals in the Eastern cultures might differ in the ability to make use of the information from the eyes and mouth based upon their sociability and personality. Therefore, we might expect that individuals who are more sociable or collectivistic are better at using information from the eyes than those who are less sociable or individualistic, which should be particularly true in those Eastern cultures that are highly collectivistic. Despite the influential stereotype that all Eastern cultures are generally less individualistic and more collectivistic than Western cultures, meta-analyses actually has shown that among Asians, only the Chinese showed consistent and large effects, that is, being both less individualistic and more collectivistic [22]. So the Chinese could be an ideal population to examine this putative correlation between individuals' collectivism/individualism tendency and their ability to use information from the eyes/mouth in interpreting others' social intentions conveyed in facial expressions (e.g., identifying real and fake smiles).

We have two specific aims in the current study. First, how the Chinese make different use of the eyes and mouth in detecting the real and fake smiles. Second, how this difference varies according to individuals' individualism and collectivism scores. Based on previous studies showing that Easterners focused much more on the eyes rather than the mouth in identification and interpretation of facial expressions, we predict that, for the Chinese, the eyes will play a more important role in successfully detection real and fake smiles than the mouth. Moreover, the ability in using information from the eyes to understand social intentions should differ between those who are more individualistic and those who are more collectivistic.

## Methods

### Participants

One hundred Chinese graduate students (50 females, *Mean age* = 23.04) participated in the study with a souvenir as payment. Five participants-were excluded from the data analysis because three had learned related smile research and two had learned related knowledge before (e.g., from the TV show “Lie to Me”). The recruitment of participants in Beijing was approved by IRBs at Beijing Normal University. Written informed consent was collected for every participant.

### Stimuli

We adapted the facial stimuli developed by Bernstein and his colleagues [23], [24]. All stimuli were located on the BBC Science & Nature Web site [25], and had been pretested for equivalency of attractiveness, trustworthiness and positivity [23], [24]. During the test, participants first rated their *Overall outlook on life* on a seven point (1–7) scale from *optimistic* to *pessimistic*, as well as *Confidence rating of your skill at discriminating between fake and real smiles*, on a seven point (1–7) scale from *low* to *high*. Then they saw twenty color video clips, each depicting an individual who had an initially neutral expression and then smiled for about 3 seconds before returning to a neutral expression again. The participants' task is to vote for each clip on whether they thought that the person's smile is *genuine* or *fake*. After they finished all 20 video clips, one more open-ended question was asked *What part of the face was most useful for discriminating between fake and real smiles?_____*. The test results were presented once the participants entered their answers to this question manually. Among the 20 individuals (7 females) who presented smiles, 10 were always exhibiting real (Duchenne) smiles and 10 were always exhibiting fake (non-Duchenne) smiles. In addition, three of them were from minority-groups, one of whom was East Asian.

Participants' individualism and collectivism scores were measured with an online version [26] of the horizontal individualism and collectivism scale [27], in which participants needed to choose on a five point (1–5) scale, whether the presented statement described them or not (from *Does not me describe at all* to *Describes me very well*). An individualism statement could be *I often do “my own thing”*, whereas a collectivism statement could be *The well-being of my coworkers is important to me* [27]. Both the individualism and the collectivism scale contained eight statements, each of which could be scored from 1 to 5, thus in total the test provided a final score ranging from 8 to 40 in both the individualism and the collectivism scale. Higher scores indicated that the person had a higher level of each value.

The two tests together took about 15 minutes, with the test order being counterbalanced between participants. Corresponding Chinese translations for both instructions and test content were done by two native Chinese speakers with sufficient proficiency in English.

### Procedure

Participants were informed that they were to perform two supposedly unrelated tasks concerning face perception and personality. All tests were conducted on a PC connected to the Internet. Each participant was tested individually, with a printed version of all the corresponding Chinese translations provided beforehand.

## Results

Based on previous studies [16], [18] and our hypothesis about the different role of the eyes and mouth in detecting and understanding smiles for Chinese, we divided our participants into two groups according to their answers to the open-ended question *What part of the face was most useful for discriminating between fake and real smiles?_____* [7]. Among those 95 valid participants, 42 entered an answer referring to the eyes region (eyes, n = 34; eye corner, n = 6; brow, n = 2), 48 entered an answer referring to the mouth region or zygomatic major muscle (mouth, n = 22; mouth corner, n = 12; zygomatic muscles, n = 6; zygoma, n = 2; cheek, n = 3; chin, n = 3), 5 entered an answer referring other regions (e.g., teeth).The final statistics were conducted on the eyes group (n = 42) and the mouth group (n = 48).

First, a Pearson's chi-square analysis was conducted to examine whether the gender of the participants differentiated the preference of the eyes and the mouth in identifying real and fake smiles. The analysis did not show significant gender difference (χ*^2^* = 2.15, *p* = 0.14). Six variables, four measured by the questionnaire items (Overall attitudes to life, Confidence of the judgment, Individualism and Collectivism) and two collected from the real and fake smiles judgment task (Accuracy and the signal detection measurement Sensitivity, *d*′ [23]), were compared between the eyes group and the mouth group, using six separate univariate ANOVAs with Age as the covariates. The Sensitivity *d*′ considers both hits (correctly identifying a Duchenne smile as genuine) and false alarms (incorrectly identifying a non-Duchenne smile as genuine) and thus examines the ability to distinguish between real and fake smiles). In our sample, it correlates strongly with the Accuracy measure (Spearman's *r* = 0.50, *p*<0.001), which confirms the internal reliability of these two measurements. The results revealed only a significant main effect of Accuracy (*F*(1,87) = 4.61, *p*<0.05), and a significant main effect of Sensitivity (*F*(1,87) = 8.26, *p*<0.01), indicating that the eyes group was significantly more accurate and sensitive (Accuracy = 70.12±13.46%; Sensitivity = 0.78±0.74) in detecting real and fake smiles than the mouth group (Accuracy = 64.58±11.05%; Sensitivity = 0.32±0.78). Such differences are also confirmed, when the eyes group was coded as 1 and the mouth group was coded as 0 [7], by a significant correlation between the eyes (vs. the mouth) group and Accuracy (Spearman *r* = 0.21, *p*<0.05), and a marginally significant correlation between the eyes group and Sensitivity (*r* = 0.30, *p*<0.01). These results are consistent with previous studies in Western cultures in which the eyes play a more important role than the mouth in distinguishing Duchenne and non-Duchenne smiles [7].

**Figure 1:**
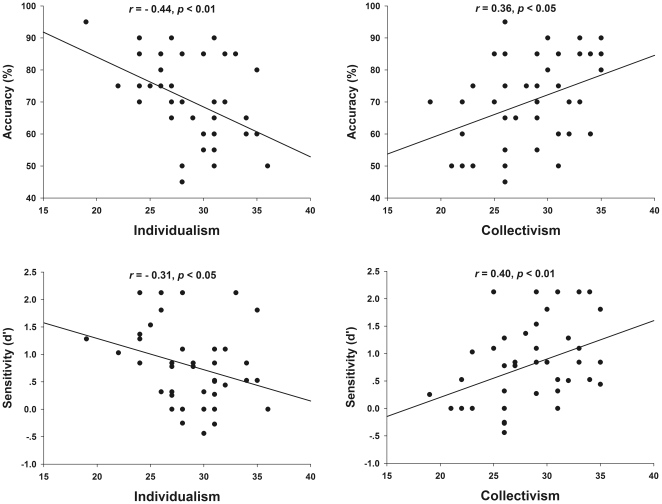
Correlations between accuracy/sensitivity and individualism/collectivism scores. Participants who preferred the eyes showed a negative correlation between accuracy and individualism scores, but a positive correlation between accuracy and collectivism scores (Top). These two correlations were also found with the sensitivity *d*′ data (Bottom). Those who preferred the mouth did not present such correlations, *ps*>0.50 (data not shown).

The influence of collectivism and individualism tendencies on individuals' smile detection strategy and performance is a central question of this study. The data showed, remarkably, for the eyes group, both the Accuracy and Sensitivity were negatively correlated with the Individualism scores (Spearman's *r* = −0.44, *p*<0.01; *r* = −0.31, *p*<0.05, respectively), and positively correlated with the Collectivism scores (Spearman's *r* = 0.36, *p*<0.05; *r* = 0.40, *p*<0.01, respectively. Fig. 1). That means, individuals with stronger collectivist tendencies are better at decoding the information from the eyes to identify and interpret others' facial expressions and social intentions; whereas those with stronger individualist tendencies are worse at using the information from the eyes. For those focus on the mouth to judge the authenticity of smiles, in contrast, their individualism and collectivism scores do not correlate with either Accuracy or Sensitivity (Individualism and Accuracy: *r* = −0.14, *p* = 0.96, Individualism and Sensitivity: *r* = 0.19, *p* = 0.20; Collectivism and Accuracy: *r* = 0.00, *p* = 0.99; Collectivism and Sensitivity: *r* = 0.16, *p* = 0.29).

Interestingly, within the eyes group, there is also a significant negative correlation between Overall attitudes to life and Collectivism scores (Spearman's *r* = −0.65, *p*<0.001), as well as a significant negative correlation between Overall attitudes to life and Confidence of the judgment (Spearman's *r* = −0.40, *p*<0.001), indicating that individuals who scored high on the collectivism scale and be more confident about their judgment generally felt more optimistic about their life than those who scored low and less confident, but only for those who focused on the eyes in interpersonal interaction.

## Discussion

In the present study, we investigated the different role of the eyes and mouth in distinguishing real (Duchenne) and fake (non-Duchenne) smiles for the Chinese, as well as how such a difference is related to individual's individualism and collectivism scores. The results showed that Chinese highly rely on information from the eyes to successfully detect the real and fake smiles, which is especially true for females, who preferred the eyes much more than males. More importantly, the ability to make use of the eyes in detecting the meaning of smiles is predicted by the individualism and collectivism scores. A higher collectivistic tendency means more accuracy and sensitivity in using the eyes to identify real and fake smiles, whereas a higher individualistic tendency means less accuracy and sensitivity in doing so. Our results therefore provide a clear empirical basis for linking individuals' personality (individualism and collectivism) with their ability of detecting and interpreting others' emotions, as well as how cultural values could impact this link by weighing the different parts of the face (e.g., the eyes and mouth) differently.

### The role of the eyes in the identification of real and fake similes

In the studies of Westerners' smiles, both the eyes and the mouth are indispensable. However, it is the mouth region that provides the most important diagnostic features for the recognition of smiles [12], [28], [29]. Studies with Westerners have found that although information in the eye region is important in identify smiles, the influence is not that remarkable. For example, a study with Europeans has found that the presence/absence of the expressive cues in the eye region (e.g., Narrowing of the eyes opening, bags and cows feet wrinkles) did not affect the accuracy of recognizing happy faces. Even smiling faces without any eye cues only slightly slowed down the reaction time than smiling faces with eye cues [8].

However, in the identification of real and fake smiles, studies with Westerners have found a more important role for the eyes. In a enjoyment (real) *vs.* nonenjoyment (fake) smiles identification task, Frank et al. [30] found that when dividing participants into three strategy groups (no quantifiable strategy, oculi strategy and zygomatic strategy) and coding the oculi group as 1 whereas the other two groups as 0, participants in the oculi group showed a positive correlation with accuracy, especially for slight intensity smiles, indicating that the eye region is crucial in distinguishing between Duchenne and non-Duchenne smiles for Westerners. Nonetheless, it is worthy to note that they did not show significant accuracy differences between different strategy groups. These shortcomings probably could be imputed to the generally weak dependence on the eyes in the identification and interpretation of smiles among Westerners [8], [12], [28], [29]. In our study with the Chinese, we found not only a similar significant positive correlation between the eyes group and their accuracy and sensitivity, but also a significant difference between the eyes and mouth groups in accuracy and sensitivity. These results thus are in line with previous studies indicating that for Easterners, the eyes play a more crucial role in detecting and interpreting facial expressions than the mouth [16], [18]. In addition, eye-tracking studies have shown that Easterners constantly ignore some critical elements of the FACS-codes faces system in identifying facial expressions, especially those around the mouth region (e.g., action units 20 (Lip Stretcher), 26 (Jaw Drop), and 27 (Mouth Stretch)) [16]. Thus our results might generalize beyond smiles to other facial expressions, and speaks to a critical issue that is active in the literature of emotional expression across cultures.

Such a crucial role of the eyes for the Chinese in interpreting others' facial expressions and social interactions was further supported by the correlation between their accuracy/sensitivity scores in distinguishing real and fake smiles and their individualism and collectivism scores (Fig. 1).

### Individualism, collectivism, and the eyes

One explanation for the reason why Easterners focus more on the eyes in the recognition and interpretation of facial expressions is that the eyes are more difficult to control than the mouth [6], [31], thus individuals from Eastern cultures, where emotional subduction is the norm, would focus more strongly on the eyes than the mouth when interpreting others' facial expressions because information conveyed by the eyes is more reliable than those by the mouth [18]. A rich body of studies (mostly with the Japanese) has suggested that Easterners are emotionally restrained [32], [33], [34], [35]. For example, Kitayama, Markus, and Kurokawa [32] demonstrated that individuals from Japanese cultural contexts report experiencing emotions less intensely than those from American cultural contexts. The reason for this, according to Markus & Kitayama [36], is because that Eastern cultures of collectivism or interdependence emphasize the indirect and implicit expression of emotions where as Western cultures of individualism or independence emphasize the direct and explicit expression of emotions.

However, none of these previous studies directly examined the connection between the role of the eyes and mouth and one's individualistic and collectivistic tendency. Our results, for the first time, not only echoed these previous findings of how individuals in a collectivistic or interdependent society could experience emotion differently according to their individualistic and collectivistic tendencies, but also provided evidence for a unique interface that connect ones personality and emotion recognition ability, that is, the eyes. Chinese participants with higher collectivistic tendency were more accurate and sensitive in using the eyes to interpret others' smiles whereas those with higher individualistic tendency were less accurate and sensitive in doing so.

Why are the eyes so important for Easterners, especially to those collectivists? One possibility is that, based upon the fact that the eyes are less controllable for the expresser, thus they might be a more reliable source of information for the observer [18]. However, the ability of using information from the eyes could differ among individuals based upon their experiences. Individuals who are more collectivistic could have more experiences on using this “trick of the eyes” in interpersonal interaction than those who are more individualistic. Studies have shown that relationships and group memberships are generally persistent and intensive for collectivists but impermanent and nonintensive for individualists [37], [38], [39], thus collectivists should have more chances to practice their interpersonal social kills than individualists.

Nonetheless, from the current results alone, it is still hard to conclude how much of the advantage of collectivists can be attributed to the experience. Further studies with more precise social personality measurements are necessary to explore this question.

### Limitations

In our study we only tested Chinese participants and looked at individual differences among them. Future cross-culture studies comparing participants from different cultures are needed to better understand if our results on the impact of individual dispositions are unique to the Chinese culture or applicable to other cultures as well.

In addition, in our study, most of the smile stimuli are presented by Western Caucasians rather than East Asians. According to the dialect theory of communicating emotion [40], [41], [42], [43], [44], [45], [46], although the language of emotion is universal, different cultures can express their emotions in dialects and thus have the potential to make the emotion recognition more accurate within cultural groups and less accurate between cultural groups, as a dialect will do in oral communication. Therefore, the different strategies we found in using the eyes and mouth might be a culturally dependent effect that only exists in Chinese or only when one judging the facial expressions from other cultural or racial groups. Further studies that directly compare the role of individualism and collectivism in smile identification in Eastern and Western cultures with stimuli from in and out-of-racial groups thus are very promising. For example, Thibault and her colleagues have conducted such a study with Quebecois, Gabonese and Mainland Chinese and found that Mainland Chinese and Gabonese do not use the Duchenne markers (e.g., the activation of the *Orbicularis Oculi* muscle) as an index of smile authenticity in a similar way as Westerners (e.g., Quebecois) do [47].

### Conclusion

How do people interpret the meaning of a smile? In the present study, we investigated the different role of the eyes and mouth in distinguishing real (Duchenne) and fake (non-Duchenne) smiles for the Chinese, as well as how such a difference is related to individual's individualism and collectivism scores. The results showed that the Chinese highly rely on information from the eyes to successfully detect the real and fake smiles. Participants who favored the eyes were more accurate than those who favored the mouth. More importantly, the ability to make use of the eyes in detecting the meaning of smiles is predicted by the individualism and collectivism scores. Higher individualistic tendency means less accurate and sensitive in using the eyes to interpret others' smiles whereas higher collectivistic tendency means more accurate and sensitive in doing so. Our results thus indicate that Chinese heavily rely on information from the eyes to identify and interpret others' facial expressions and social intentions.
